# Supercritical Fluid Processing of Polymers

**DOI:** 10.3390/polym11101551

**Published:** 2019-09-24

**Authors:** Stefano Cardea, Ernesto Reverchon

**Affiliations:** Department of Industrial Engineering, University of Salerno, Via Giovanni Paolo II, 132, 84084 Fisciano (SA), Italy; ereverchon@unisa.it

The use of supercritical fluids instead of organic solvents has attracted the interest of numerous researchers, due to the unique peculiarities of supercritical fluids which are characterized by solvent powers comparable to those of liquid organic solvents, diffusivity comparable to those of gaseous substances and quasi-zero surface tension. As a consequence, numerous "traditional" processes have been improved by the use of supercritical fluids, overcoming their limitations. Among them, the processes concerning the use and/or transformation of polymers are certainly becoming more and more attractive. Indeed, polymers have numerous fields of applications, ranging from the chemical industry to the pharmaceutical industry, from the food industry to tissue engineering, from mechanical engineering to computer engineering. Thus, the interaction between polymers and supercritical fluids has become crucial in the recent scientific literature. For example, numerous micronization processes, porous structures generation processes (aerogels, membranes, scaffolds, foams), fiber and film production processes, impregnation processes, etc., have been developed using supercritical fluids.

This special issue currently consists of a total of nine articles written by research groups of experts in the field. The papers can be substantially divided into two main topics:

- polymeric structure (nanoparticles, microparticles, foams) formation [1,2,3], useful for drug release applications;
- effects of supercritical fluids on polymer characteristics [4,5,6,7,8].

An example of purification by supercritical fluids has been also published [9].

Regarding the polymeric structure formation, in the study of Jia et al. [1], berberine-loaded solid polymeric particles (BPs) with β-cyclodextrin (β-CD) were generated using a solution-enhanced dispersion process assisted by supercritical fluids (SEDS). Moreover, the relationship between dissolution and berberine (BBR) bioavailability was evaluated. The most interesting result was that the dissolution property was controlled by adjusting the operating parameters during the SEDS process. Characterizations on BP indicated that BBR was dispersed in amorphous form, while methoxy groups of BBR were included in the cavities of β-CD. In vivo pharmacokinetic studies showed that oral bioavailability increased by about 54% and 86% when the dissolution rate of BBR was increased by 51% and 83%, respectively. The entry speed of BBR into the bloodstream was also advanced with the degree of dissolution enhancement. It seemed that dissolution enhancement had a positive effect to the oral bioavailability of berberine. Meanwhile, the authors suggested that the supercritical carbon dioxide (SC-CO_2_)-assisted technology is a promising method for pharmaceutical research, due to its advantages in regulating drug-dosage properties.

A new process assisted by SC-CO_2_ that can largely improve classical electrospray (ESPR) atomization for the formation of polymeric micro and nanoparticles was proposed by Baldino et al. [2]. The consequent reduction of surface tension and viscosity due to the SC-CO_2_ addition allows the production of polymeric micrometric or nanometric particles of controlled size and distribution at a production rate up to one hundred times that of the traditional process. The new process was applied to particle generation from a very high molecular weight polyvinylpyrrolidone (PVP) and tested at different polymer percentages by weight and at different pressures. The results were very promising. Indeed, repeatable microparticle diameters and distributions were obtained, ranging between 0.55 and 2.25 µm at PVP concentrations from 1% to 5% w/w and pressures between 80 and 120 bar. This new process is a serious candidate to substitute the traditional electrospray process in the next years.

Foaming of polycaprolactone impregnated with quercetin was studied by García-Casas et al. [3]. The process was carried out with a batch foaming technique using SC-CO_2_. The experimental design was developed to study the influence of pressure (15–30 MPa), temperature (308–333 K), and depressurization rate (0.1–20) on the foam structure, melting temperature and release tests of composites. It was observed that the porosity created in the polymer had a heterogeneous structure, as well as the impregnation of the quercetin during the process. On the other hand, controlled release tests showed a significant delay in the release of quercetin compared to commercial quercetin. Furthermore, the SC-CO_2_ assisted process was confirmed to be a valid alternative to the traditional process, concerning the generation of polymeric structures for drug release applications.

Concerning the effect of supercritical fluids on polymers characteristics, Qiao et al. [4] studied the effect of the temperature on the structures and properties of PAN fibers cyclized in SC-CO_2_. The PAN fibers processed in the SC-CO_2_ confirmed that the degree of cyclization increased with the increase of the cyclization temperature. Compared with the PAN fibers treated in the air, the PAN fibers treated in SC-CO_2_ showed a higher degree of cyclization even at the same temperature. These findings might be related to the osmotic action of SC-CO_2_ causing the fibers to be further arranged in a regular manner, which was favorable for the cyclization reaction. Moreover, as one kind of high diffusion and high heat transfer media, the heat release during the cyclization of PAN fibers could be quickly removed by SC-CO_2_, which achieved the progress of the rapid-entry cyclization reaction. The same authors, in another work [5], studied the effect of different pressures of SC-CO_2_ on crystallinity, degree of orientation and mechanical property of PAN fibers during the hot-drawing process. The results showed that as the pressure increased, the crystallinity, and degree of orientation of PAN fibers increased. Furthermore, when the pressure was 10 MPa, the crystallinity increased from 69.78% to 79.99%, which was the maximum crystallinity among the different pressures. However, when the pressure was further increased, the crystallinity and degree of orientation of the fibers were reduced. The test results of the mechanical properties were consistent with the trends of crystallinity and degree of orientation, showing that when the pressure was 10 MPa, the tensile strength of the fibers increased from 4.59 to 7.06 cN·dtex^−1^ and the modulus increased from 101.54 to 129.55 cN·dtex^−1^.

In a related work, Xiaoma et al. [6] studied the effect of SC-CO_2_ on Aramid fibers (AFs), in particular, on a kind of AFs: F-III fibers. In order to obtain F-III fibers with high mechanical properties, pristine F-III fibers were hot drawn at the temperature of 250 °C, pressure of 14 MPa, tension of 6 g·d^−1^, and different times, which were 15, 30, 45, 60, 75, 90, and 105 min, respectively, in SC-CO_2_. All the samples, including the pristine and treated F-III fibers, were properly characterized. The results showed that the thermal stability of F-III fibers was enhanced to some extent, and the tensile strength and modulus of F-III fibers had great changes as the extension of treatment time during hot drawing in SC-CO_2_, although the treatment temperature was lower than the glass transition temperature (T_g_) of F-III fibers. In conclusion, the hot drawing in SC-CO_2_ was successfully applied to the preparation of F-III fibers with high mechanical properties.

The effect of SC-CO_2_ on high modulus AF, such as Kevlar 49, was studied by Kong et al. [7]. This fiber is conventionally prepared by the heat annealing of high strength aramid fiber under a suitable tension at high temperature, especially higher than 500 °C. This enables the mobility of a rigid molecule chain to be rearranged into a more perfect crystalline or orientation structure under tension. However, annealing decreases the tensile strength, since the thermal degradation of the molecular chain at a high temperature cannot be avoided. Kevlar 49 fibers treated in SC-CO_2_ under tension could improve their mechanical properties at a low temperature. The effects of the tension on the mechanical properties and structure of the Kevlar 49 fibers were studied: The results show that the mechanical properties, crystallinity, and orientation of the fiber can be improved when the tension is less than 0.6 cN·dtex^−1^, which may be due to the increase of the mobility of a rigid segment with the help of the plasticization of SC-CO_2_ and re-arrangement of macromolecular chain into crystalline and orientation structure under tension. The amorphous region also was enhanced by crosslinking reaction of toluene 2,4-diisocyanate (TDI) with the chain end groups of the macromolecules in the amorphous regions. However, a decrease of tenacity was found when the tension was higher than 0.6 cN·dtex^−1^, which is because the tension was so high that the microfibril was broken. The results indicated that treating the Kevlar 49 fiber in SC-CO_2_ under a suitable tension with TDI as a crosslink agent can simultaneously improve both the tenacity and modulus of the fiber.

Modeling of the mechanical properties of porous polymers processed by SC-CO_2_, using a phenomenological approach was proposed by Tabernero et al. [8]. Tensile and compression tests of alginate/gelatin and cellulose acetate/graphene oxide were modeled using three hyperelastic equations, derived from strain energy functions. The proposed hyperelastic equations provide a fair good fit for mechanical behavior of the nanofibrous system alginate/gelatin (deviations lower than 10%), whereas, due to the presence of the solid in the polymer network, a four-parameter model must be used to fit the composite cellulose acetate/graphene oxide behavior. Larger deviations from the experimental data were observed for the system cellulose acetate/graphene oxide because of its microporous structure. A finite element method was, then, proposed to model both systems. It allowed a realistic description of observable displacements and effective stresses. The results indicate that materials processed using SC-CO_2_, when submitted to large stresses, do not obey Hooke’s law and must be considered as hyperelastic.

A SC-CO_2_ assisted purification process was studied by Yu et al. [9]. In particular, the cyclic oligomers in the highly chemically resistant polyester polybutylene terephthalate (PBT) were effectively removed using an SC-CO_2_ antisolvent technique in which 1,1,1,3,3,3-hexafluoro-2-propanol (HFIP) was used as the solvent. In addition to the oligomers, tetrahydrofuran was completely removed because of its low molecular weight and liquid state. The effects of the operating variables, including temperature, pressure, and the PBT concentration in HFIP, on the degree of removal of the oligomers were systematically studied using experimental design and the response surface methodology. The most appropriate operating conditions for the purification of PBT were 8.3 MPa and 23.4 °C when using 4.5 wt % PBT in HFIP. Under these conditions, the cyclic trimers and dimers could be removed by up to 81.4% and 95.7%, respectively, in a very short operating time.

In conclusion, the articles published in this special issue, and reported above, confirmed the interesting peculiarities of supercritical fluids, i.e., SC-CO_2,_ that allow to improve the traditional processes performance [1,2,3,9] and the characteristics of a large number of polymers [4,5,6,7,8].

In particular, an innovative SC-CO_2_ assisted electrospray process has been proposed for the first time [2], showing promising results in terms of polymeric particles generation, and two different SC-CO_2_ assisted techniques (SEDS and SC-foaming) for the generation of polymeric drug-loaded devices have shown interesting results in terms of drug controlled release [1,3], i.e., modifying the processes parameters it is possible to control the specific drug release. Moreover, SC-CO_2_ confirmed the capability of improving the mechanical characteristics of several polymers (i.e., polymeric fibers) at specific operating conditions [4,5,6,7,8].
